# More Than Meets the Ear: Incidental Cerebrospinal Fluid Leak During Myringotomy in a Child With Common Cavity Deformity

**DOI:** 10.7759/cureus.113318

**Published:** 2026-07-24

**Authors:** Alan Wu, Ajay Bharathan, Parth Nakirikanti, Santino S Cervantes

**Affiliations:** 1 College of Medicine, University of Central Florida College of Medicine, Orlando, USA; 2 Pediatric Otolaryngology, Nemours Children's Hospital, Orlando, USA; 3 Otolaryngology, University of Central Florida College of Medicine, Orlando, USA

**Keywords:** cerebrospinal fluid leak, cochlear malformation, common cavity deformity, congenital ear malformation, myringotomy, pediatric otolaryngology

## Abstract

Cerebrospinal fluid (CSF) otorrhea represents a breach of the temporal bone separating the subarachnoid space from the middle ear and carries a significant risk of meningitis. While most cases are associated with trauma or prior skull base surgery, congenital fistulas may remain occult until unmasked by otologic procedures.

We report the case of a five-year-old male with bilateral profound sensorineural hearing loss and previously unknown labyrinthine anomalies who developed high-flow, pulsatile clear otorrhea during routine left ear myringotomy for recurrent otitis media. Immediate concern for CSF leak prompted fluid collection for beta-2 transferrin analysis and urgent surgical management. Subsequent exploration via lateral temporal bone resection revealed a congenital inner ear fistula with active CSF egress from the vestibule through the oval window. The leak was successfully managed using a multilayer closure technique including vestibular packing with temporalis fascia, cartilage reinforcement, Eustachian tube obliteration, pedicled temporalis muscle flap coverage, and blind sac external auditory canal closure.

This case illustrates the recognition and acute intraoperative management of an unanticipated high-flow CSF leak during a routine pediatric otologic procedure, and supports preoperative temporal bone imaging in children with profound sensorineural hearing loss before middle-ear instrumentation. Untreated, such leaks carry a significant risk of life-threatening meningitis.

## Introduction

Cerebrospinal fluid (CSF) otorrhea, the escape of CSF through the ear, represents a critical defect in the integrity of the temporal bone [1,2]. While often secondary to trauma or skull base surgery, the incidence of spontaneous CSF otorrhea is increasingly recognized, particularly in patients with predisposing factors such as elevated intracranial pressure or anatomic problems like tegmen tympani (the roof of the middle ear) bone defects [3,4].

A routine myringotomy, typically performed to manage middle ear effusion, then serves as the critical inciting event. By creating a direct communication between the fluid-filled middle ear and the external environment, the procedure converts a contained or nasally draining leak into an active otologic fistula, resulting in persistent, clear watery drainage [5]. This active leakage not only poses a significant burden to the patient but, more importantly, establishes a direct pathway for infection, increasing the risk of potentially fatal meningitis [6].

We present the case of a five-year-old boy with bilateral profound sensorineural hearing loss and no prior imaging in whom a routine left myringotomy for recurrent otitis media unexpectedly produced a high-flow, pulsatile CSF otorrhea, ultimately attributed to a common cavity deformity with a fistula from the vestibule through the oval window. CSF leakage from congenital inner ear malformations, including its precipitation by myringotomy or ventilation tube placement, is a recognized phenomenon [5,7]. The value of this report lies less in the rarity of the entity itself than in the unexpected intraoperative scenario it highlights: a high-flow leak discovered during a routine procedure in a child without prior meningitis or an established diagnosis, requiring immediate temporary measures before definitive repair could be planned. We use this case to highlight (1) the recognition and acute intraoperative management of unexpected high-flow CSF otorrhea, and (2) the rationale for preoperative temporal bone imaging in children with profound sensorineural hearing loss before middle-ear instrumentation.

## Case presentation

**Figure 1:**
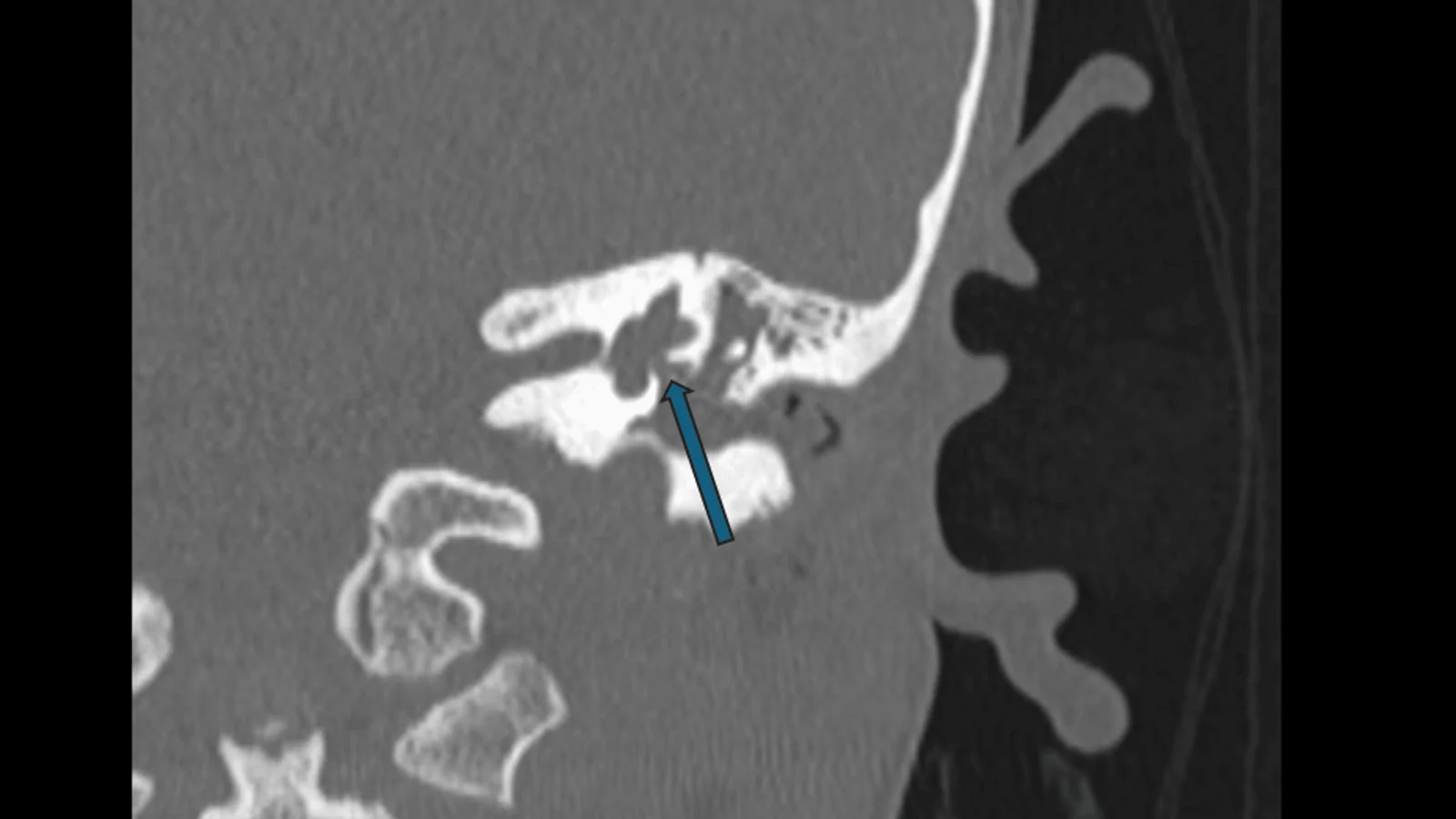
Coronal CT of the left temporal bone demonstrating a cystic cochleovestibular malformation (common cavity deformity) with a focal bony deficit connecting the cystic cavity to the middle ear space (blue arrow). This abnormal communication corresponds to the pathway through which CSF was found to egress intraoperatively from the vestibule through the oval window.

**Figure 2:**
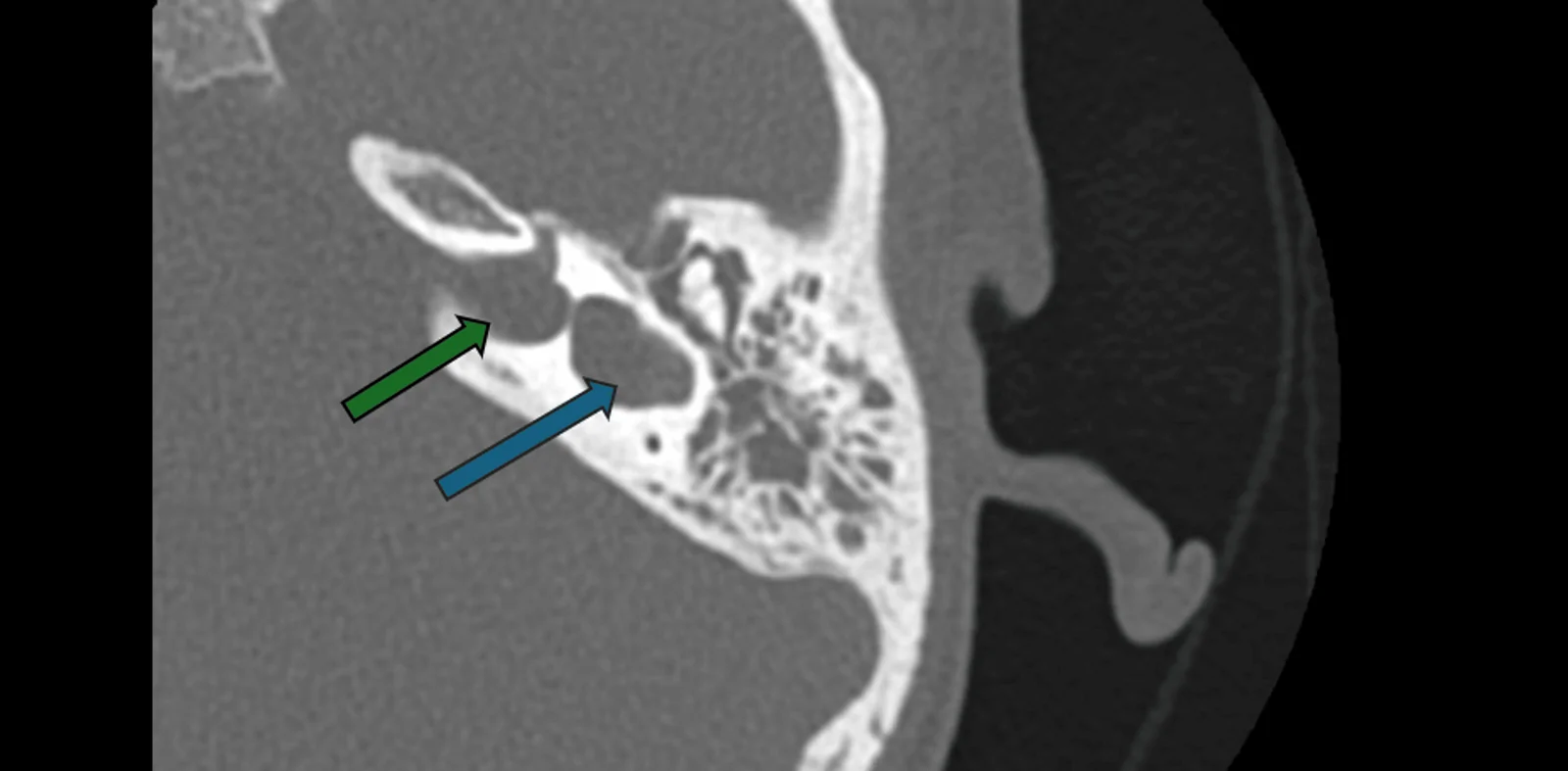
Axial CT of the left temporal bone showing a bulbous, widened internal auditory canal (green arrow) and a common cavity deformity replacing the normal cochlear and vestibular architecture (blue arrow). These findings are consistent with common cavity deformity and explain the congenital fistulous connection that permitted high-flow CSF leakage into the middle ear.

Patient familial consent was received for this case report. The patient was a five-year-old male with bilateral profound sensorineural hearing loss and no prior imaging studies. He also had a history of chronic left-sided clear rhinorrhea that had not previously been recognized as CSF. During a myringotomy to address recurrent otitis media, a cut was made in the usual inferior location in the left tympanic membrane. Clear fluid rapidly filled the ear canal and was suctioned. The fluid was continuously emitted in a pulsatile fashion from the incision despite repeated suctioning. A CSF leak was suspected and 2 mL of fluid was collected to be tested for beta-2 transferrin. It was then decided to decrease intracranial pressure and to close the myringotomy locally to prevent additional leakage and stabilize the patient for further assessment. Using an endoscope, a butterfly biodesign graft was deployed to close the perforation, but CSF continued to leak. A fibrin sealant was then implemented, followed by a gelatin sealant, and then additional fibrin sealant, which seemingly contained the leak. This was confirmed by performing a gentle Valsalva maneuver. The patient was then handed off to the anesthesia service and transferred to the PICU intubated. Given the patient’s abnormal CSF otorrhea, a labyrinthine anomaly was suspected and imaging was ordered. A CT scan of the left ear showed a large cystic cavity within the otic capsule with absence of internal bony architecture, dysmorphic semicircular canals, and an enlarged internal auditory canal, consistent with a common cavity deformity (Figures 1, 2). 

Ten days later, a left lateral temporal bone resection was performed to identify and address the underlying source of the CSF otorrhea. The surgical course was initiated with standard posterior ear canal incisions, leading to the postauricular exposure of the mastoid cortex. The critical intraoperative finding occurred immediately upon elevation of the tympanomeatal flap, revealing a copious volume of clear, pulsatile drainage. This finding, which was inconsistent with typical middle ear effusion, confirmed a high-flow CSF leak, with fluid samples collected for definitive beta-2 transferrin analysis. 

To achieve adequate visualization of the temporal bone floor and roof, the medial external auditory canal (EAC) skin and tympanic membrane were resected, followed by removal of the incus and a partial mastoidectomy with resection of the bony EAC. Inspection of the most common site for spontaneous leaks, the tegmen tympani, confirmed that it was intact, shifting the diagnostic focus. The presence of malformed semicircular canals further indicated a congenital inner ear anomaly. After skeletonizing the tympanic and the proximal mastoid facial nerve, the origin of the leak was precisely identified. Inspection of the oval window revealed high-flow CSF egressing from the vestibule through a congenital dehiscence in the oval window/footplate region, consistent with the underlying inner ear malformation. The stapedius tendon was then divided and the stapes footplate removed to fully expose the vestibule for packing.

The high-flow nature of the leak was managed with a multilayer closure technique. Temporalis fascia was harvested and packed tightly into the vestibule. This primary seal was augmented with a supportive cartilage graft placed over the promontory, stabilizing the packing beneath the malleus. To prevent CSF from being forced back up the Eustachian tube, its orifice was permanently occluded with a deep plug of sternocleidomastoid muscle. A Valsalva maneuver was performed to confirm a watertight seal. The final deep layer consisted of an absorbable collagen-based matrix patch placed over the medial temporal bone, secured with fibrin sealant. For protection, a pedicled temporalis muscle flap was rotated over the entire defect and secured to the surrounding fascia. Finally, the external auditory canal was permanently closed with a blind sac closure to eliminate any remaining pathway for fluid drainage or ascending infection. Postoperatively, the CSF otorrhea resolved, as did the patient's longstanding clear rhinorrhea. At follow-up, there was no recurrence of otorrhea or rhinorrhea and no episode of meningitis.

## Discussion

This case illustrates the acute intraoperative recognition and management of an unanticipated high-flow CSF leak unmasked by routine myringotomy in a child with a previously undiagnosed congenital inner ear malformation. The precipitation of CSF otorrhea from inner ear malformations by myringotomy or ventilation tube placement is well described [5,7], but most reported cases are identified after one or more episodes of meningitis or in children already known to harbor a labyrinthine anomaly. The present case is distinguished by the absence of any prior meningitis or established diagnosis and by antecedent clear rhinorrhea that had not been recognized as CSF, such that the high-flow otorrhea presented as an abrupt intraoperative event. We therefore focus on the practical decision-making this scenario demands.

When high-flow clear otorrhea appears unexpectedly during myringotomy, the immediate priorities are to halt the procedure, avoid suctioning that may enlarge the fistula, collect fluid for beta-2 transferrin, and to temporize the leak while follow-up care is arranged [8]. In this patient, endoscopic placement of a butterfly biodesign graft followed by sequential fibrin-gelatin-fibrin sealant achieved a temporary seal, after which intracranial pressure was lowered and the child was intubated and transferred to the PICU. This staged strategy, starting with acute temporization, then imaging, and then planned definitive repair ten days later, permitted both anatomic characterization and a controlled reconstruction, rather than emergent exploration of an undiagnosed malformation.

Congenital inner ear malformations, including incomplete partition anomalies and other dysplastic labyrinthine configurations, can result in abnormal communications between the subarachnoid space and the inner ear. In this particular case, the malformation was a common cavity deformity, a severe congenital anomaly in which the cochlea and vestibule fail to differentiate and are represented by a single cystic cavity lacking internal bony architecture, typically associated with profound sensorineural hearing loss [9]. Depending on the malformation, CSF or perilymph may track into the middle ear through several routes, including the oval window, the round window, the internal auditory canal along the facial nerve, the hypotympanum, or preformed fissures such as the Hyrtl fissure [6,7]. Although an intact tympanic membrane can keep such leaks clinically silent, in this patient it instead directed CSF down the Eustachian tube, so that the fistula manifested as chronic rhinorrhea rather than otorrhea until the myringotomy created a direct otologic route. That CSF had been draining chronically by the nasal route indicates the fistula was already active before any otologic intervention; a leak brisk enough to egress by both nasal and, once the tympanic membrane was breached, otologic routes underscores its high-flow nature. This dual egress also compounded the risk of ascending infection, since the child was already exposed through the nasopharynx before the external auditory canal provided a second pathway. A problem like this is associated with significant morbidity and mortality, largely attributable to its elevated risk of meningitis [6,10]. In a cohort with persistent CSF rhinorrhea, Daudia et al. reported a meningitis risk of approximately 19% (0.3 episodes per patient-year) prior to definitive repair; comparable rates of around 20% are described for spontaneous CSF otorrhea [10,11].

Beta-2 transferrin analysis remains the gold standard for biochemical confirmation of CSF leakage due to its high specificity for CSF compared with serum or middle ear effusion. In a study done by Bhat et al., beta-2 transferrin demonstrated a pooled sensitivity of 96% and specificity of 94.6% for detecting CSF leaks [12]. Prompt collection of fluid in this case was appropriate and aligned with established diagnostic algorithms. However, the high-flow nature of the leak and the patient’s congenital anatomy necessitated early surgical intervention rather than conservative management.

Intraoperative exploration demonstrated that the tegmen tympani, the most common site of spontaneous CSF leaks in adults, was intact [4]. Instead, the leak originated from the vestibule through a congenital oval-window/footplate defect, further confirming the diagnosis of a common cavity deformity. This finding is consistent with prior reports describing congenital vestibular fistulas associated with a malformed cochleovestibular structure and profound sensorineural hearing loss [13,14].

The definitive approach must be tailored to leak location, flow rate, and hearing status. Recent literature has emphasized minimally invasive repair of inner ear-derived CSF leaks, including transcanal endoscopic stapedectomy with vestibular packing and transmastoid vestibule obliteration, with good outcomes in appropriately selected patients [5,7]. In the present case, several factors favored a more extensive approach: the leak was high-flow and had already proven refractory to initial endoscopic temporizing measures. The leak originated from the vestibule through a congenital oval-window/footplate defect in a young child with a labyrinthine anomaly, and since hearing was defective bilaterally, a blind-sac canal closure and Eustachian tube obliteration added no further functional compromise while closing residual escape pathways and mitigating the risk of ascending infection. We therefore performed a lateral temporal bone resection with multilayer closure. This included packing the vestibule with temporalis fascia, reinforcing the repair with cartilage, obliterating the Eustachian tube, placing a collagen-matrix dural substitute, covering the area with a pedicled temporalis muscle flap, and closing the external auditory canal as a blind sac. This approach has been previously described for spontaneous otogenic CSF leaks in children [15]. We emphasize that this extensive reconstruction reflects the specific high-flow, non-serviceable-hearing context and should not be regarded as the default, as in many patients a minimally invasive approach is appropriate and preferable.

This case reinforces the need for heightened preoperative suspicion in children with congenital hearing loss undergoing middle ear procedures. Review of imaging, awareness of anatomic risk factors, and preparedness for intraoperative CSF leak management are essential to prevent catastrophic complications. This case supports the consideration of preoperative temporal bone imaging before middle ear procedures in pediatric patients with sensorineural hearing loss, particularly when congenital ear anomalies may be present.

## Conclusions

This case underscores the importance of recognizing congenital inner ear malformation as a potential cause of CSF otorrhea in pediatric patients. Although myringotomy is a routine and generally safe procedure, it may unmask a previously unrecognized CSF fistula in children with underlying labyrinthine anomalies, resulting in high-flow leakage and significant risk of meningitis. Prompt intraoperative recognition, confirmatory testing, and definitive surgical repair are critical to achieving favorable outcomes. Successful management in this case required a multilayered reconstructive approach combined with permanent external auditory canal and Eustachian tube closure. Surgeons performing middle-ear procedures in children with profound sensorineural hearing loss should be aware of this condition, consider preoperative temporal bone imaging when appropriate, and be prepared to identify and temporarily manage an unexpected high-flow leak during surgery.
